# Dynamic Variation of Ecosystem Services Value under Land Use/Cover Change in the Black Soil Region of Northeastern China

**DOI:** 10.3390/ijerph19127533

**Published:** 2022-06-20

**Authors:** Quanfeng Li, Lu Wang, Guoming Du, Bonoua Faye, Yunkai Li, Jicheng Li, Wei Liu, Shijin Qu

**Affiliations:** 1 School of Public Administration and Law, Northeast Agricultural University, Harbin 150030, China; quanfeng.li@neau.edu.cn (Q.L.); s211202037@neau.edu.cn (L.W.); a12190433@neau.edu.cn (Y.L.); a03180211@neau.edu.cn (J.L.); a12190202@neau.edu.cn (W.L.); 2 Land Remote Sensing Big Data Technology Innovation Center, Harbin 150030, China; 3 School of Economics and Management, Northeast Agricultural University, Harbin 150030, China; bonoua.faye2021@neau.edu.cn; 4 School of Public Administration, China University of Geosciences, Wuhan 430074, China; qusj@cug.edu.cn

**Keywords:** land use/cover change, ecosystem services value, dynamic variation, black soil region, northeastern China

## Abstract

A better understanding of the dynamic variation in the ecosystem service value (ESV) under land use/cover change (LUCC) is conductive to improving ecosystem services and environmental protection. The present study took Landsat TM/ETM remote sensing images and socio-economic statistic data as data sources and extracted land-use data using RS and GIS technology at 5-year intervals from 1990 to 2020. Then, we interpreted the spatio-temporal characteristics of LUCC and analyzed ESV changes using the value equivalence method in the black soil region of northeastern China (BSRNC). The main results showed that land use changed significantly during the study period. Cultivated land continued to expand, especially paddy areas, which increased by 1.72 × 10^6^ ha, with a relative change of 60.9% over 30 years. However, grassland decreased by 2.47 × 10^6^ ha, with a relative change of −60.6% over 30 years. The ESV showed a declining trend, which decreased by CNY 607.96 million during 1990–2020. The decline in forest and grassland caused a significant decline in the ESV. Furthermore, the ESV sensitivity coefficients were less than one for all of the different categories of ecosystem services. LUCC has a considerable impact on ESV in the BSRNC, resulting in ecosystem function degradation. As a result, future policies must emphasize the relationship between food security and environmental protection in situations of significant land-use change.

## 1. Introduction

Ecosystem services refer to services that affect human well-being and that are derived from the ecosystem on the Earth’s terrestrial surface [1,2]. Hence, the links between man and nature are closely related and may even be inseparable. As such, this interconnection implies repercussions for ecosystem functioning. Previous studies have highlighted that human manipulation and the degradation of the environment have reached the point in which the cumulative global impacts affect our food, fiber, and water systems and threaten people’s health and well-being [3]. However, the population growth issues that are connected with socioeconomic development may reflect the contradiction between human activities and human well-being [4]. Under this context, ecosystem service value (ESV) is indispensable when balancing the need for food production and ecological protection because it can clarify the ecological assets and values provided by a region [5].

Additionally, changes in the functioning of the Earth’s ecological systems can pose a significant threat to human survival. In this situation, implementing suitable land and ecosystem services are essential carriers of terrestrial ecosystems. Land-use change also affects regional ecosystem service functions by changing the distribution of regional resources and ecosystem types. The landscapes of ecological systems have been considerably affected by ongoing land use/cover change (LUCC), which represents the biggest element of global change. In addition to the background noted earlier, another study shed light on that fact that since the mid-20th century, 60% of global ecosystem services have been degraded due to irrational land utilization [6]. This mode of land use has appeared in different ways in different regions. This scenario has resulted in desertification, deforestation, climatic change, and decreased carbon sequestration [7,8]. This situation is visible in large agricultural countries [9,10], especially China. Within this region, the ecosystem service value (ESV) is a significant indicator of the ecosystem function changes caused by LUCC [11,12,13]. Therefore, analyzing the dynamic variation in ESV under LUCC has become a widespread issue. A detailed comprehension of the variability in the ESV may potentially improve the performance of global environmental policies.

ESV approaches have been used to evaluate ecosystem services on a global and regional scale [14,15,16]. In that sense, ESV assessment may provide a momentous reference for ecosystem protection [17]. Costanza et al. [14] first proposed analyzing ESV changes on a global scale. Due to the tempo-spatial heterogeneity, this study cannot be directly applied to the national scale, especially in China. Xie et al. [16] revised the equivalent factor to establish the national scale spatio-temporal dynamic assessment method, resulting in different regional conditions showing unique ecosystem services and functions [18,19]. Nonetheless, this method has been improved. The evaluation method and ESV equivalence table have been further revised based on existing studies and factors [20].

Many studies have revealed that land-use dynamics can cause ESV change [9,21]. LUCC may increase ecosystem supply or cause ecological degradation [22]. For instance, population growth and food demands have driven the transfer of cultivated land to grassland [23,24]. The rapid shrinkage of forest and grassland areas has led to the degradation of ecosystem functions. In this situation, it is essential to analyze the relationship between ecosystem functions and land use to better understand how LUCC affects ecosystem functions. However, existing land and ecological policies ignore assessing and reflecting the hidden ecological value and cost of land use and rarely consider the relationship between land use and ecosystem services [25,26]. 

In China, the government has launched ecological restoration and protection policies to improve ecosystem function [27,28,29]. Previous research has shown that establishing ecological restoration and conservation policies enhances ecosystem services and functions [30,31]. Some studies have also neglected to cover the loss in the ESV caused by the lack of land-use policies implemented at the regional level [32]. Hence, the sustainable development of ecological functions and systems requires a comprehensive analysis of the variations in LUCC and ESV on a large spatial scale. With the development of remote sensing and GIS technologies, the quantitative analysis of LUCC and ESV has become more accessible [33]. In this work, we estimated the ESV and evaluated the relationship between LUCC and the ESV, providing data reference that can be used by regional governments to coordinate the interaction between land-use policies and ecosystem services. Some researchers have shown that the ESV often varies in terms of its temporal and spatial characteristics [34,35]. For example, Shang et al. found that the interaction between the NDVI and soil types had the most substantial impact on the spatial differentiation of the IESV in the study area, with an explanatory power of 21.95% [5]. Therefore, it is essential to carry out some studies in different regions to analyze changes in the ESV.

The black soil region of northeastern China (BSRNC) is one of the four black soil regions in the world. This area represents the area with the largest amount of grain production in China, accounting for 1/4 of the country’s total grain output [36,37]. The BSRNC has evolved from natural forest and grassland ecosystems to artificial farmland ecosystems [38]. About 70% of the land categories have shown changes that have resulted in deforestation, soil, and water loss [39]. Irrational land use has led to decreases in the fertility of black land and the degradation of ecosystem services. Primarily, cultivated land expansion has placed tremendous pressure on the ecosystem. The government has established ecological restoration and protection measures to enhance ecosystem functions, such as supporting the sustainable development of prominent grain-producing regions and land conservation tillage planning [40]. Although these measures have been implemented to restore and protect ecosystems, the ESV has rarely been assessed. Furthermore, the variation in the ESV under LUCC is not clear in this vital region. To some extent, the lack of data and information on the spatio-temporal changes resulting from LUCC hinders black land protection and ecological restoration processes. Therefore, an accurate study outlining the rules of ESV variation under LUCC plays a strategic role in promoting environmental conservation and natural resource utilization in the BSRNC.

This paper takes the whole BSRNC as its study area to research ESV changes caused by LUCC from 1990 to 2020. The objectives of this study are as follows: (1) analyze the temporal and spatial variation caused by LUCC in the BSRNC; (2) revise the equivalence factor to calculate the ESV; And (3) evaluate the credibility of the research results. To address the objectives, we first introduce the study area and describe the source of the data and process them. Then, we use the dynamic degree index of land utilization to quantitatively analyze the variation in the temporal and spatial characteristics of LUCC. Furthermore, the study used an equivalence factor to determine the ESV related to the physical geography of the BSRNC and then assessed the results. Following this, we discuss the relationship between the ESV and LUCC and present a policy to protect ecosystem function and the ecological environment. Finally, we put forward conclusions and directions for future studies.

## 2. Materials and Methods

### 2.1. Study Area

**Figure 1:**
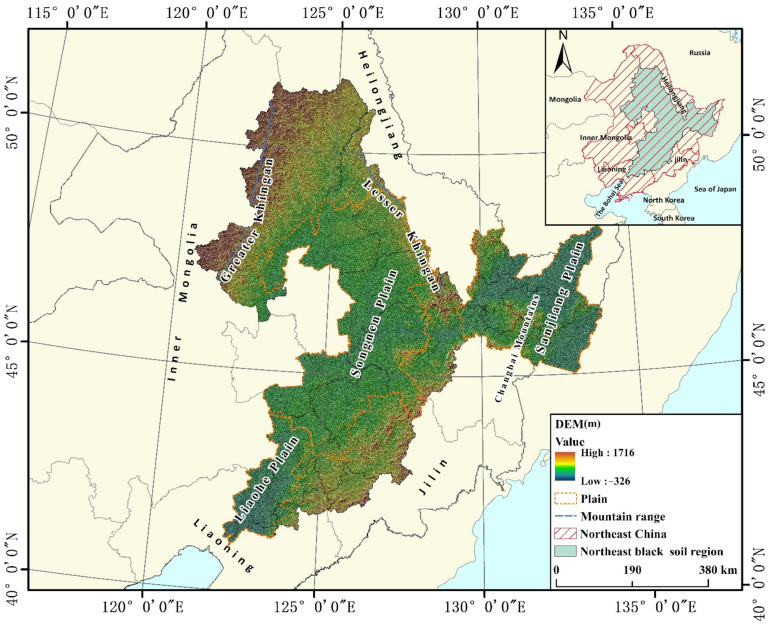
Study area.

The BSRNC is located between the latitudes of 41°04′–51°37′ N and the longitudes of 120°30′–134°06′ E (Figure 1). The topography conditions in this area are complex, surrounding the Greater Khingan, Lesser Khingan, and Changbai Mountains. The regional landform mainly comprises hills and mountains distributed in the Sanjiang, Songnen, and Liaohe Plains. The BSRNC belongs to the temperate-continental monsoon climate zone and has average annual precipitation ranging from 500–700 mm/year. Furthermore, rainfall is mainly concentrated in the crop-growing season, from April to September, accounting for 80% to 90% of the annual precipitation. Suitable and superior natural geographical conditions promote the stable development of regional agriculture, making the BSRNC a substantial grain base [38]. The BSRNC plays a key role in China’s ecological conservation and food security [41]. However, the growing human population has significantly prompted LUCC in the region, resulting in a reclamation rate in the BSRNC of about 75%. Additionally, unsustainable human land-use practices contribute to the degradation of cultivated land quality in black soil significantly, threatening national food security. Biodiversity, nutrient maintenance, hydrological networks, and other ecological services all suffer as a result of long-term and spatial reclamation. Therefore, we chose the BSRNC as the research area because it is significant for national food security and we want to strengthen ecological protection in this area.

### 2.2. Data Sources

This study acquired the 1:100 million spatial distribution data of Chinese soil types from the Data Center for Resources and Environmental Sciences, Chinese Academy of Sciences (RESDC) (http://www.resdc.cn, accessed on 16 April 2022). We extracted the spatial distribution data of black soil from the spatial distribution data on soil types. This paper determined the location of the BSRNC according to the list outlining the protection and utilization of black land and the principle of centralized contiguous land (Figure 1). Landsat TM/ETM remote sensing images were the source of the LUCC data used in this study. As such, the spatial resolution was 30 × 30 m, and the comprehensive accuracy was higher than 93% [42,43]. 

This paper obtained land-use type data from the Chinese remote sensing monitoring database of land-use status. In the BSRNC, cultivated land (paddy and dry land) accounts for the highest area. The mixed coniferous broad-leaved, broad-leaved, and bush forest types are the main components of the forest ecosystem. The grassland ecosystem is dominated by high grassland coverage. The water area classification includes rivers, lakes, tidal flats, and beaches. According to the land source characteristics in the BSRNC and the results of a secondary ecosystem classification by Xie et al. [44], we divided the land-use types into six categories: paddy, dry land, forest, grassland, water area, and unused land. The grain output and area data were from the 2010 and 2020 editions of the Heilongjiang Statistical Yearbook, Liaoning Statistical Yearbook, and Jilin Statistical Yearbook. The grain price data were sourced from China’s 2020 Agricultural Price Survey Yearbook [45].

### 2.3. Methods

LUCC dynamics alter the variations in the ESV, which can cause the degradation of ecosystem services [9,21]. This paper takes LUCC as the breakthrough point to study the changes in the ESV in the BSRNC from 1990 to 2020. We interpreted the land use dynamic index and land use dynamic degree to quantitatively analyze the temporal and spatial variation characteristics of LUCC. We quoted the unit price of food production service and value coefficient (VC) of farmland to calculate the ESV and quantitatively analyze the variation in its spatio-temporal characteristics. Then, the sensitivity coefficients of various land-use types were determined to verify the feasibility of the evaluation results. Further, we revealed the regularities in LUCC based on physical geographical location to lay an essential foundation to further explore ESV changes in the BSRNC. Then, according to Xie et al. [15,16] and the actual situation in the BSRNC, this paper does not consider the changes in construction land and does not involve the calculation of its ESV.

#### 2.3.1. Quantitative Calculation of LUCC

This study analyzed LUCC by comparing the areas occupied by each land-use type during the study period. We calculated total area gains and losses across each land-use type. In this article, we chose two indices, the land use dynamic index and land use dynamic degree [25], to analyze LUCC in the BSRNC using the following equation [46,47]:

(1) $$D=\frac{U_{y}-U_{x}}{U_{x}}\times \frac{1}{T}$$

where *D* refers to the dynamic degree of a certain land-use type; *U_y_* is the area of a certain land-use type at the end of the study period; *U_x_* is the area at the beginning of the study period; *T* is the year during the research period.

#### 2.3.2. Measurement of the ESV

a. Equivalent factor correction

In this article, the equivalent factors for different land-use types were determined according to Xie et al.’s 2017 study [16] and the geographical characteristics of the BSRNC. Scholars have also used Xie’s method to revise the equivalent factor when conducting studies in northeast China [48]. The study used the data collected by other scholars to correct the equivalence factor. Because the farmland comprising the study area is basically paddy fields and dry land, the equivalence of the paddy field and dry land factors correspond to those for paddy and dry land in Xie et al. [16]. The forests in the study area include the coniferous, mixed coniferous broad-leaved, broad-leaved, and bush forest types. Therefore, the forest factor is the average value of coniferous, mixed coniferous broad-leaved, broad-leaved, and bush forest factors. Grassland basically is prairie, so the grassland factor adopts the value of the prairie factor. The water area factor is the value of water. Most of the unused land is bare land, and the unused land factor adopts the value of the bare land factor. This research examined whether physical geographical factors influence changes in ecological land use. As a result, the changes in the construction land area and ESV are not considered in this paper. The equivalence factors used in this study are shown in Table 1.

b. Unit Price of Food Production Service on Cultivated Land

This article revised the ESV of the cultivated land ecosystem per unit area by referring to the research results of Xie et al. [16]. Meanwhile, the equivalence factor of the ESV per unit area was defined as being equal to 1/7 of the average economic value of the grain production of cultivated land. The formula is as follows:

(2) $$E_{a}=1/7\sum_{i=1}^{n}\frac{m_{i}p_{i}q_{i}}{M},i=1,2\ldots n$$

where *E_a_* is the economic value of grain production per unit area of cultivated land; *i* is crop species; *p_i_* is the average price of the crop *i* (RMB/kg); *q_i_* is the yield per unit area of the crop *i* (kg/ha); *m_i_* is the sowing area of the crop *i* (ha); and *M* is the sown area of all crops(ha).

This study chose rice, maize, and soybean as the main crops and counted their yield, sowing area, yield per unit area, and price in 2020, which was finally calculated to be CNY 2433.86/ha.

c. Value coefficient

To determine the equivalent value coefficient, we referred to Costanza et al. [1,14] and Xie et al. [15]. We adopted the value coefficient to calculate the ESV per unit area of different land-use types. It was calculated as follows:

(3) $$VC_{ij}=e_{ij}\times E_{a},i,j=1,2\ldots n$$

where *VC_ij_* refers to the unit price of the ecological service *i* of ecosystem *j* (CNY/ha); *i* refers to the type of ecological service function; *j* refers to the ecosystem type; *e_ij_* provides the equivalence factor of the unit price of the ecological service *i* in ecosystem *j*.

d. Regional *ESV*

Based on the land-use data from 1990 to 2020 and the *VC* of different land types, we calculated the *ESV* in the BSRNC to provide a data reference for ecosystem restoration. The calculation formula of *ESV* is as follows [14]:

(4) $$ESV=\sum A_{k}\times VC_{k},ESV=\sum A_{k}\times VC_{k}{}_{f}$$

In the formula, *ESV* represents the ecosystem service value of non-construction land. *A_k_* is the distribution area of land-use type *K* in the study area. *VC_k_* is the ecosystem service value coefficient per unit area of land-use type *K*. *VC_kf_* is the value of the ecological service function of land-use type *K*. 

#### 2.3.3. Sensitivity Analysis

Sensitivity analysis was used to certify the elasticity between the total *ESV* and *VC* of different land-use types, representing a significant portion of *ESV* evaluation [9]. The economic concept of the elasticity coefficient was used to calculate the sensitivity of the *ESV* to determine the dependence of the *ESV*. The specific calculation equation is as follows [9,49,50]:

(5) $$CS=\left|\frac{(ESV_{n}-ESV_{m})/ESV_{m}}{(VC_{nl}-VC_{ml})/VC_{ml}}\right|$$

where *CS* is the sensitivity coefficient. *ESV_m_* and *ESV_n_* are the initial and adjusted *ESV*, respectively. *VC_ml_* and *VC_nl_* are the initial and adjusted *VC*, respectively.

If *CS* is greater than 1, it indicates that the *ESV* is elastic to the *VC* and that the research results have poor accuracy and low credibility. On the contrary, if *CS* is less than one, it means that *ESV* is inelastic to *VC* and that the research results are credible [51,52,53].

## 3. Results

### 3.1. Land-Use Change and Dynamics

#### 3.1.1. Temporal Land-Use Changes

Land use in the BSRNC changed significantly from 1990 to 2020. Cultivated land (paddy and dry land) and forestland were the dominant land-use types in the BSRNC. These two land-use types represented more than 80% of the total area. Out of all of the land-use types, cultivated land accounted for the largest proportion of the total area coverage (>48%), followed by forestland (35%), while water area, grassland, and unused land account for a smaller proportion of area coverage.

**Figure 2:**
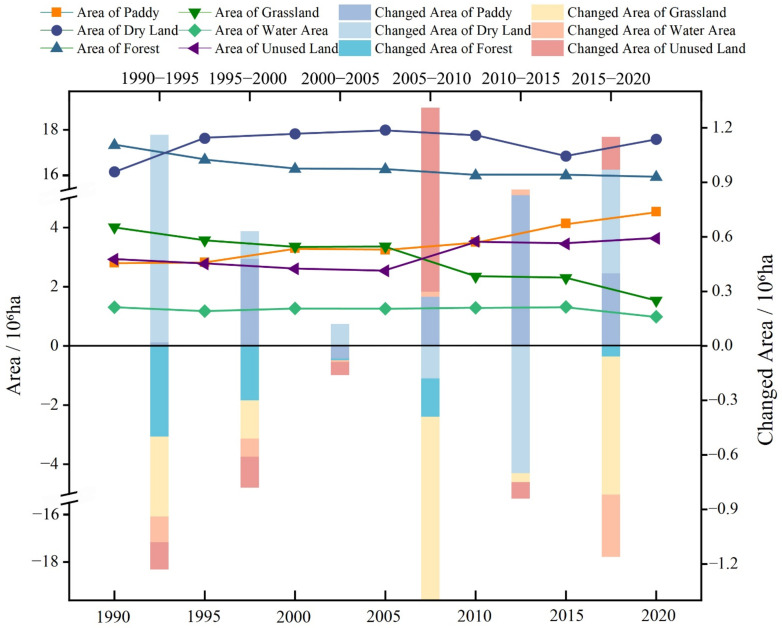
Temporal variations in land-use in the BSRNC.

Figure 2 shows that the changes that took place between land-use types in the study area were complex. From 1990 to 2020, the paddy land area increased continuously from 2.80 × 10^6^ ha to 4.52 × 10^6^ ha. The grassland land area exhibited a continuous decline during the study period, from 4.01 × 10^6^ ha to 1.54 × 10^6^ ha, decreasing by 2.47 × 10^6^ ha. In particular, grassland area decreased sharply in 2005–2020 and 2015–2020, from 3.36 × 10^6^ ha to 2.35 × 10^6^ ha and from 2.30 × 10^6^ ha to 1.54 × 10^6^ ha, respectively. During the whole study period, dry land significantly increased from 16.58 × 10^6^ ha to 17.68 × 10^6^ ha, increasing by 1.10 × 10^6^ ha. In the same sense, from 1990 to 1995, dry land increased from 16.58 × 10^6^ ha to 17.72 × 10^6^ ha, increasing by 1.14 × 10^6^ ha. Following this trend, we noted that dry land increased from 17.11 × 10^6^ ha to 17.68 × 10^6^ ha during the period of 2015–2020, increasing by 0.57 × 10^6^ ha. 

From 1990 to 2020, the forest land area declined significantly, from 17.48 × 10^6^ ha to 16.40 × 10^6^ ha, decreasing by 1.08 × 10^6^ ha. As for water, land area showed a slowly declining trend during the study period. The overall change was not obvious, and its area decreased by 0.32 × 10^6^ ha. Unused land showed a slowly increasing trend from 1990 to 2020. With the exception of the period from 2005 to 2010, the changes in unused land were not more obvious than they were in other years. Among all of the land-use types, unused land accounts for a very small proportion, representing only 0.08% of the total.

Overall, essential transitions happen between cultivated land, forestland, and grassland. Due to large-scale reclamation, more forestland and grassland are converted into cultivated land. Thus, the increase in cultivated land is the largest in the BSRNC. The percentage of cultivated land increased from 41.6% in 1990 to 48.7% in 2020, increasing by 7.1%. The forest and grassland decreased significantly. The percentage of forestland decreased from 37.5% in 1990 to 35.2% in 2020.

#### 3.1.2. Land-Use Spatial Change

**Figure 3:**
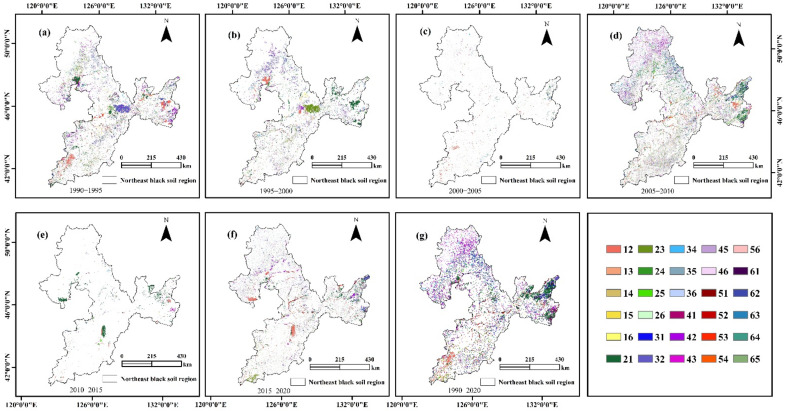
Spatial distribution of land-use transfer in the BSRNC from 1990 to 2020. Notes: 1: Paddy; 2: dry land; 3: forest; 4: grassland; 5: water area; 6: unused land. (**a**) is the spatial distribution of land-use transfer in the BSRNC from 1990 to 1995; (**b**) is the spatial distribution of land-use transfer in the BSRNC from 1995 to 2000; (**c**) is the spatial distribution of land-use transfer in the BSRNC from 2000 to 2005; (**d**) is the spatial distribution of land-use transfer in the BSRNC from 2005 to 2010; (**e**) is the spatial distribution of land-use transfer in the BSRNC from 2010 to 2015; (**f**) is the spatial distribution of land-use transfer in the BSRNC from 2015 to 2020; (**g**) is the spatial distribution of land-use transfer in the BSRNC from 1990 to 2020.

Notable land use/cover conversions were identified between 1990 and 2020 (Figure 3). As depicted in Figure 3, cultivated land (paddy and dry land) expansion was clearly observed across all of the BSRNC during the study period. However, extensive deforestation was observed in the five geographic regions. Most of the forest types were lost in the Greater and Lesser Khingan Mountains and in the Songnen Plain. In contrast, cultivated land expansion occurred in the Liaohe Plain, Sanjiang Plain, and Songnen Plain. Moreover, during the research period, the spatial distribution of grassland changed dramatically due to grassland expansion in the Sanjiang Plain, which replaced cultivated land. Unused land became distributed throughout the study area.

Additionally, a large amount of forest was converted to cultivated land in the Songnen Plain from 1990 to 1995. Then, the conversion of dry land to paddy is significant. This conversion was mainly concentrated in the Sanjiang Plain from 1995 to 2000. The main drivers for this were to ensure food security policies and respond to climate change. From 2005 to 2010, a direct conversion of forest and unused land to cultivated land was noted in the Sanjiang Plain. From 2015 to 2020, dry land and paddy conservation were found in the Songnen Plain. During this period, paddy field expansion was the result of increasing demands for food due to population growth.

#### 3.1.3. Land-Use Dynamic Changes

Several significant changes in the dynamic degree of land-use can be observed in the whole study area (Table 2). An overall increasing trend can be observed in paddy, dry land, and unused land, with paddy land increasing the most, up to 1.72 × 10^6^ ha, with the rate of change being 60.9% and a dynamic degree of 2.03%. However, forest, grassland, and water area show a decreasing trend. Grassland decreased the most, dropping by −2.47 × 10^6^ ha, with the rate of change being −60.6% and the dynamic degree being −2.02%. Forest decreased by 1.08 × 10^6^ ha, with the rate of change being −6.18% and the dynamic degree being −0.20%.

Overall, the paddy land area increased by 60.9% from 1990 to 2020, in contrast to a minor decrease between 2000 to 2005 (−1.99%). The highest paddy change dynamic (18.03%) was observed between 2010 to 2015. This increase could have been attributed to the country’s policies targeted at enhancing food security. Grassland decreased significantly from1990 to 2020 (−60.6%), while a slight increase was witnessed from 2000 to 2005 (0.21%). In the forest, there seems to be a slight decreasing trend of −6.18% from 1990 to 2020. 

Additionally, the change trends in the water land area and unused land are fundamentally opposed to one another. The water land area decreased by −24.65%, and the unused land area increased by 24.08% during the study period. From 2015 to 2020, the relative change in the grassland and water land area was the most significant, changing by −33.12% and −25.25%, respectively. This situation showed that because of the rising prices of agricultural products and the government’s agricultural development policies, local governments and agricultural producers continue to impact ecological land in order to develop cultivated land due to the multiple incentives of economic benefits. 

### 3.2. Variations of ESV

#### 3.2.1. Temporal Variation Analysis of ESV

From 1990 to 2020, the total ESV showed negative growth in the BSRNC. As shown in Table 3, the total ESV decreased from CNY 14,792.86 million to CNY 14,184.90 million. In fact, during the study period, forest land had the highest ESV (>57%). However, unused land contributed to the total ESV the least, contributing about 0.001%. The ESV provided by cultivated land has increased continuously over the past 30 years, from CNY 1883.59 million to CNY 2154.35 million. The proportion of arable land increased from 12.7% to 16.3%.

However, the ESV of forest and grassland decreased significantly, from CNY 8407.49 million to CNY 7887.65 million and from CNY 494.61 million to CNY 189.45 million, respectively. The results showed that the cultivated land area experienced an obvious increase during the study period. The ecosystem’s unit area value was moderate, and it could not counterbalance the decrease in the region’s ESV induced by forest and grassland losses. The ESV of water area and unused land fluctuated from 1990 to 2020. The ESV of water area fell by CNY 57.15 million, whereas the ESV of unused land grew by CNY 3.43 million.

Only paddy, dry land, and unused land showed a growing trend in the ESV among the six land categories that were investigated. The ESVs of other land-use types fell, particularly those of grassland and water area, indicating that the ecosystems in the BSRNC are under tremendous downward pressure and require further adjustments.

#### 3.2.2. ESV Spatial Variation Analysis

From 1990 to 2020, the amount and distribution of land types in the BSRNC changed considerably, which had a significant impact on the spatial change in the ESV. In particular, cultivated land expansion impacted the spatial distribution changes in the ESV.

**Figure 4:**
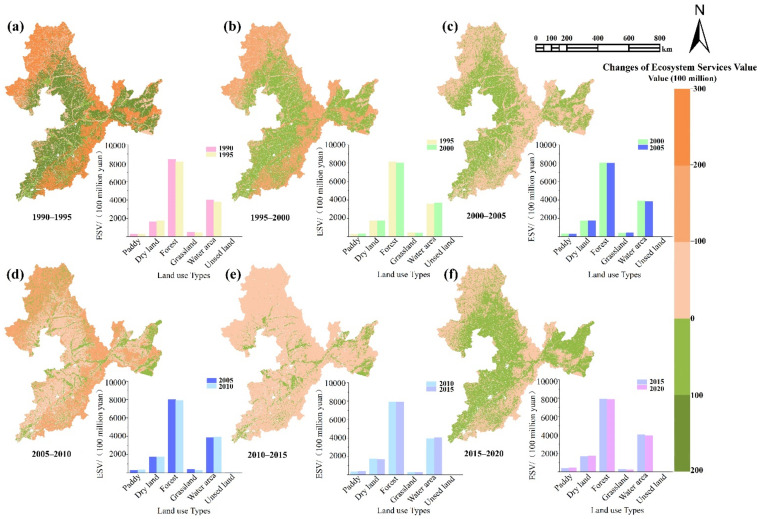
Spatial variation of the ESV in the BSRNC. Notes: The subfigures represent the change in ESV for different land use types.

As shown in Figure 4, we found that the area with the most significant decline in the ESV was located in the Greater and Lesser Khingan Mountains and Changbai Mountains, and this was mainly because of the massive forest land loss in these mountains. The expansion of cultivated land led to a decline in forest area in the region—numerous forest land types with high-value equivalence coefficients result in a high ESV. However, the cultivated land low-value equivalence coefficients result in a low ESV. The increase (CNY 163.35 million RMB) in the ESV caused by the expansion of arable land cannot compensate for the decrease (-CNY 519.84 million) in the ESV caused by the decrease in the forest area. As a result, the ESV in these mountain regions has been declining, seriously affecting the ecosystem services and ecosystem functions in these regions.

From 1990 to 2005, the ESV continuously increased in the Sanjiang Plain and Liaohe Plain, which was mainly due to cultivated land. In 2005, the implementation of returning farmland to forest and grassland restricted the conversion of forest into cultivated land, which enabled the ESV of the region to experience negative growth. After 2015, implementing the national food security strategy and establishing high-standard farmland resulted in the positive growth of the cultivated land ecosystem.

#### 3.2.3. Value Analysis of Various Land Ecosystem Service Functions

Table 4 shows the changes in the ecosystem services for various land types. Regulation services contributed the most to the proportion of the ESV, while cultural services accounted for the lowest proportion of the total ESV from 1990 to 2020. In addition, a continuously increasing trend was documented in regulation services, while provision, support, and culture services showed fluctuating changes in the past 30 years. 

The highest to lowest proportion of the service value of the land ecosystem for each year comprises hydrological regulation, climate regulation, gas regulation, soil formation and retention, biodiversity, environmental purification, food production, aesthetic landscape, raw material production, water supply, and nutrient cycling. The ESV of hydrological and climate regulation accounted for more than 50% of the total ESV. From 1990 to 2020, continuous increases were found in food production, increasing from CNY 577.35 million to CNY 638.36 million. The value of other ecosystem service functions decreased at different rates. Hydrological regulation experienced a significant drop of CNY 836.46 million during the study period. From 2015 to 2020, there was an obvious decrease in the water supply that was determined to be closely related to the rapid decline of the forest and grassland areas.

Meanwhile, the expansion of cultivated land in the BSRNC is essential for food production, but this expansion leads to ecosystem destruction. A continuous decline was observed in the water supply, gas regulation, climate regulation, environmental purification, and hydrological regulation. Climate regulation dropped from CNY 2817.18 million in 1990 to CNY 2601.94 million in 2020. Environmental purification dropped from CNY 988.97 million in 1990 to CNY 886.60 million in 2020. A series of data shows that the expansion of cultivated land leads to a decline in the ESV.

### 3.3. Sensitivity Coefficient Analysis

The Coefficient of Sensitivity (CS) was used to evaluate the sensitivity and consistency of our ESV estimations. As such, the assessed ecosystem service value for 1990 to 2020 is presented in Table 5. This result shows different aspects, and there are various land-use types.

The sensitivity coefficients of the ESV to the VC for diverse land-use types were less than one. In fact, from 1990 to 2020, water area recorded its highest CS of 0.82% in 2020. During the same period, the VC of unused land was 0.01%. Land-use types such as paddy and dry land, which represent high-land-use area, incurred a moderate value. Paddy land recorded a maximum value of 0.35% in 1990. Alternatively, the value VC of dry land remains the same over the years at 0.06%.

Consequently, if we consider the order of the degree of sensibility, we can note the following order: water area > grassland > paddy > forest > dry land > unused land. This context demonstrated that the ESV in the BSRNC lacked elasticity to VC and that the results were reliable. In conclusion, the analysis revealed that the paddy VC declined significantly from 0.36% in 1990 to 0.22% in 2020, a differential of −0.14%. Alternatively, the VC of the water area increased from 0.77% in 1990 to 0.82% in 2020.

## 4. Discussion

### 4.1. Interpretation of LUCC in the BSRNC

Over the past 30 years, a large number of forest and grassland areas have been replaced with cultivated land. The area of cultivated land increased from 19.38 × 10^6^ ha in 1990 to 22.21 × 10^6^ ha in 2020, increasing by 3.83 × 10^6^ ha. There have changes between dry land and paddy areas, most of which involve the conversion of dry land to paddy. The paddy land area in the BSRNC continued to expand from 2.80 × 10^6^ ha to 4.52 × 10^6^ ha, increasing by 1.72 × 10^6^ ha during the study period. Mao et al. [40] have shown that China has also implemented a series of policies to promote agricultural development and grain production. For example, China launched a food security project to increase grain production and ensure food security in the face of a rapidly growing population [23].

Additionally, the implementation of conversion from dry land to paddy has caused wetland and grassland loss and degradation, which may lead to the problem of water shortage [54]. Natural resources, such as water, may have a significant influence on this land transition, notably paddy expansion for increased food production. We found that there were abundant water resources to increase the paddy area in the BSRNC, which has an average surface runoff of 2634.3 × 10^8^ m^3^/year and precipitation of 567.5 × 10^8^ m^3^/year [36]. In this context, it is evident that water supply resources provide favorable conditions for agricultural production, which can also explain the expansion of paddy land area during the study period. Meanwhile, climate data from meteorological stations in the colder areas of China displayed that the air temperature warming rate was 0.1 °C/decade during 1986–2015, with intensified warming (0.2 °C/decade) after the 2000s. Climatic warming increased the accumulated temperature during the rice growing season and stimulated the climate adaptability of crops in this region [55]. Under these factors, grain production has increased rapidly, but the sustainable utilization of resources and the effective management of ecosystems face serious challenges.

Intense LUCC has a negative impact on the ecosystem and ecological environment. China developed larger-scale ecosystem restoration policies, particularly in the BSRNC. These policies include the Grain for Green Policy and the Natural Forests Conservation Policy [30,56]. Although many ecosystem restoration policies have been implemented, the improvement of ecosystem services still needs more time [57]. Hence, analyzing the relationship between the ESV and LUCC is indispensable for ecologically sustainable development.

### 4.2. The Relationship between ESV and LUCC

Many studies have shown that LUCC significantly affects changes in the ESV [39,58]. In particular, irrational land-use change can lead to ecosystem degradation. Many studies have used the CS to evaluate the sensitivity and consistency of their ESV estimations. As such, this study used the CS to assess the relationship between the ESV and LUCC. Based on the results of the sensitivity analysis, the sensitivity coefficients of ESV and VC for different land-use types were less than one. These results demonstrated that LUCC is closely related to ecosystem services in the BSRNC.

The intrinsic relationship between humans and natural systems was effectively shown by examining the LUCC process and its associated consequences on ecosystem services. Furthermore, the expansion of the cultivated land area to promote food production has led to a significant decrease in hydrological, climate, and gas regulation and as well as a decrease in the total ESV. Our results indicate that between 1990 and 2020, the ecosystem providing the ESV decreased by CNY 607.96 million. The significant increase in food production during the study period caused paddy expansion. Because of the high demand for water in paddy areas, the water supply has shrunk, decreasing by CNY 187.56 million. Projects to increase the grain production capacity and to improve rice yield in order to promote food grain have also led to forest and grassland degradation, leading to a significant decrease in climate regulation, environmental purification, hydrological regulation, and soil formation and retention. In the BSRNC, forest areas decreased substantially, especially due to the conversion to cultivated land, and global warming benefits from the reclamation of cultivated land [59]. There is also a need to feed the increasing population [41]. The conversion from forest land to other land-use types has contributed to local ESV loss the most. Analyzing these trends raises the question of whether it is possible to compare ecosystem services over time. Since ecosystem services are related to the value society assigns to the goods and services produced by nature, the exact delivery of a service might be valued quite differently over time. To avoid these problems, we focused on comparing the potential of the land-use pattern to provide ecosystem functions.

In this study, the decrease in the ESV is mainly due to the expansion of cultivated land, which resulted in forest and grassland losses. The two land types are considered significant providers of ecosystem services. Large-scale deforestation and reclamation have led to the deterioration of the ecosystem, which has a seriously impact on the ecological environment in the Greater Khingan Mountains and Lesser Khingan Mountains. With the Songnen Plain and Sanjiang Plain at the core of grain output, the region has focused on developing modern agriculture and expanding cultivated land. Notably, after 2005, agricultural producers have still continued to impact ecological land to develop cultivated land due to the government’s agricultural development policy in the Sanjiang Plain. Hence, the deterioration of ecological land leads to reductions in regional natural resources and the degradation of ecosystem services such as climate regulation, gas regulation, water supply, and biodiversity.

LUCC significantly drives variations in the ESV at the national scale [60,61,62]. Ouyang et al. [30] researched improved ecosystem services from 2000 to 2010 at the country scale and found that ecosystem services were distinctive in different regions due to spatial heterogeneity and diverse policies [63,64]. Rapid socioeconomic development and policy changes may accelerate LUCC or transfer land into a new type. Concurrently, many developed and developing regions are experiencing a decline in cultivated land and an increase in urban land [51,65,66]. Therefore, many scholars focus on the relationship between urban land expansion and ESV [60,66]. However, BSRNC, a major grain-producing area in China, is mainly manifested in the relationship between the disorderly expansion of cultivated land and ESV. In terms of the transition of forest and grassland to cultivated land, this transfer facilitates the rapid decline in the ESV, which may have a negative impact on the regional ecological environment and system.

### 4.3. Regional Land-Use Policy Implications for Ecological Protection

The BSRNC represents important forest resources and a grain production base, with large-scale reclaimed forest accounting for over 1000 ha since 1990 [33]. Land consolidation projects and deforestation activities have significantly increased the amount of potential cultivated land in the BSRNC. As a result of these measures, cultivated land has rapidly expanded while forest and grassland have reduced significantly, resulting in a substantial impact on ecosystems. This is related to the government’s implementation of several major programs after the year 2000, including the Grain for Green project and the natural forest protection project [30,56]. The implementation of these ecological projects has significantly improved the ecological environment but has also led to declines in the cultivated land area. From 2000 to 2005, the decreased arable land accounted for 63.5% of the total land in China [67]. In this context, food security has once again become a core concern of the Chinese government. In 2010, China implemented the grain production capacity construction project to increase grain production and ensure food security. The local government recognized the Songnen and Sanjiang Plains as being vital to modern agriculture development. Even though the government has adopted several legislations to safeguard ecosystems, ecological conservation still faces significant challenges.

The regional functional orientation determines regional land-use change. Therefore, the conflict between food production and ecological protection leads to changes in the relative importance of cultivated land and ecological land resources in different periods and then changes the relative scarcity of different types of land resources. As the prominent grain-producing area in China, the BSRNC should emphasize the security of grain yield and how to increase it and should pay attention to regional ecological security as well as strengthen the ecological support for food security, rationally plan the ecological resources of different land types, protect the ecological land, and establish a dual assessment mechanism for food security and ecological security.

The decrease in the ESV varies in different regions, so the government should optimize the spatial patterns of the main functional zones for food and ecological security according to the actual regional conditions. In the ecological zones for the Greater Khingan Mountains and Lesser Khingan Mountains, land resources should be well protected, and the ecological balance should be effectively maintained. Additionally, comprehensive management should be carried out to ensure the coordinated development of agricultural production and the environmental environment. This will result in improved grain production in the Sanjiang Plain and Songnen Plain and leads down the road to sustainable agricultural development.

In summary, the synchronization of ecosystem conservation strategies is hindered by our lack of understanding regarding variation in the ecosystem service value and fundamental laws, spatiotemporal changes in biophysical conditions, and ecosystem structures. As such, for above this scenario, future LULC policies should pay more attention to balancing the relationship between agricultural land expansion and ecosystem services.

## 5. Conclusions

In this article, we analyzed the variations in the ESV under LUCC in the BSRNC from 1990 to 2020 using a value equivalence method. The results showed that land use in the BSRNC changed significantly from 1990 to 2020. Cultivated land continued to increase from 19.38 × 10^6^ ha to 22.21 × 10^6^ ha. However, grassland in the BSRNC continued to decrease, decreasing from 4.01 × 10^6^ ha to 1.54 × 10^6^ ha. Cultivated land such as paddy areas continued to increase, from 2.80 × 10^6^ ha to 4.52 × 10^6^ ha, increasing by 1.72 × 10^6^ ha. Policy implementation is a crucial factor affecting the expansion of paddy areas and of the conversion of dry land into paddy areas. The ecosystem resulted in the ESV decreasing by CNY 607.96 million during the study period. Cultivated land, forest, and water body areas were the main land-use types that made the greatest contributions to the ESV. The change trends observed in the ESV for land use in the BSRNC are consistent with the changes in its main components. Then, the evaluation results of the ESV proved to be reliable due to the sensitivity indexes being less than one (<1). The sensitivity coefficients followed water body > grassland > paddy > forest > dry land > unused land in 2020, which indicates that the land-use changes lack flexibility in the changes in the ESV.

This study can provide more effective policy-making and data support for ecosystem restoration and environmental protection in China’s black land region. However, the research still has some limitations. We did not study the temporal and spatial changes of construction land or calculate its ESV. We also only discussed the impact of LUCC on the ESV and did not consider the impact of other factors on the ESV, such as urbanization. Consequently, to improve the accuracy of the assessment results, future studies should focus on the spatial-temporal changes in the ESV that are driven by both natural and economic factors. Moreover, the driving factors resulting in ESV changes in the BSRNC should also be considered in future research.

## Figures and Tables

**Table 1:** Equivalence value per unit area of the ecological service of non-construction land in the BSRNC.

Primary Types	Secondary Types	Paddy	Dry Land	Forest	Grassland	Water Area	Unused Land
Provision	Food production	1.36	0.85	0.25	0.10	0.80	0.00
	Raw material production	0.09	0.40	0.58	0.14	0.23	0.00
	Water supply	−2.63	0.02	0.30	0.08	8.29	0.00
Regulation	Gas regulation	1.11	0.67	1.91	0.51	0.77	0.02
	Climate regulation	0.57	0.36	5.71	1.34	2.29	0.00
	Environmental purification	0.17	0.10	1.67	0.44	5.55	0.10
	Hydrological regulation	2.72	0.27	3.74	0.98	102.24	0.03
Support	Soil formation and retention	0.01	1.03	2.32	0.62	0.93	0.02
	Nutrient cycling	0.19	0.12	0.18	0.05	0.07	0.00
	Biodiversity	0.21	0.13	2.12	0.56	2.55	0.02
Culture	Aesthetic landscape	0.09	0.06	0.98	0.25	1.89	0.01
Total		3.89	4.01	19.76	5.07	125.61	0.20

**Table 2:** Evolution of dynamic land-use types in the period of 1990–2020 (%).

Periods		Land-Use Types					
		Paddy	Dry Land	Forest	Grassland	Water Area	Unused Land
1990–1995	Relative change	0.71	6.85	−2.85	−10.91	−10.18	−5.28
	Dynamic degree	0.14	1.37	−0.57	−2.18	−2.04	−1.06
1995–2000	Relative change	16.92	0.84	−1.81	−6.00	7.99	−6.04
	Dynamic degree	3.38	0.17	−0.36	−1.20	1.60	−1.21
2000–2005	Relative change	−1.99	0.72	−0.06	0.21	−0.77	−2.59
	Dynamic degree	−0.40	0.14	−0.01	0.04	−0.15	−0.52
2005–2010	Relative change	8.16	−1.01	−1.25	−30.27	2.38	39.49
	Dynamic degree	1.63	−0.20	−0.25	−6.05	0.48	7.90
2010–2015	Relative change	18.03	−3.92	−0.01	−2.13	2.30	−2.53
	Dynamic degree	3.61	−0.79	0.00	−0.43	0.46	−0.51
2015–2020	Relative change	9.66	3.33	−0.34	−33.12	−25.25	5.28
	Dynamic degree	1.93	0.67	−0.07	−6.62	−5.05	1.06
1990–2020	Relative change	60.90	6.64	−6.18	−60.60	−24.65	24.08
	Dynamic degree	2.03	0.22	−0.20	−2.02	−0.81	0.79

**Table 3:** Ecosystem service values of different land-use types from 1990 to 2020 (CNY 100 million).

Year Land-Use Types	Quantity	Paddy	Dry Land	Forest	Grassland	Water Area	Unused Land
1990	ESV	265.38	1618.21	8407.49	494.61	3992.94	14.23
1995	ESV	267.26	1729.06	8167.85	440.65	3786.28	13.48
2000	ESV	312.47	1743.64	8020.02	414.21	3672.91	12.66
2005	ESV	306.24	1756.10	8015.32	415.06	3842.91	12.34
2010	ESV	331.22	1738.33	7915.14	289.41	3934.34	17.21
2015	ESV	390.94	1670.07	7914.26	283.25	4024.91	16.77
2020	ESV	428.73	1725.62	7887.65	189.45	3935.79	17.66
1990–1995	Variation	1.88	110.85	−239.64	−53.96	−206.66	−12.25
1995–2000	Variation	45.21	14.58	−147.83	−26.44	−113.37	−0.82
2000–2005	Variation	−6.23	12.46	−4.70	0.85	170	−0.32
2005–2010	Variation	24.98	−17.77	−100.18	−125.65	91.43	4.87
2010–2015	Variation	59.72	−68.26	−0.88	−6.16	90.57	−0.44
2015–2020	Variation	37.79	55.55	−26.61	−93.85	−89.12	0.89
1990–2020	Variation	163.35	107.41	−519.84	−305.21	−57.15	3.43

**Table 4:** ESV of different categories of ecosystem services from 1990 to 2020 (CNY 100 million).

Primary Types	Secondary Types	1990	1995	2000	2005	2010	2015	2020
Provision	Food production	577.35	594.82	613.15	613.38	615.18	622.04	638.36
	Raw material production	435.31	437.14	435.09	436.02	428.58	423.13	424.31
	Water supply	227.62	195.58	181.34	183.57	169.13	134.28	40.06
Regulation	Gas regulation	1234.42	1222.32	1222.38	1222.10	1204.98	1210.43	1213.34
	Climate regulation	2817.18	2736.48	2699.93	2698.46	2640.03	2642.42	2601.94
	Environmental purification	988.97	948.54	948.34	946.57	934.32	938.41	886.60
	Hydrological regulation	5233.60	4855.47	5088.16	5059.46	5107.62	5217.08	4397.14
Support	Soil formation and retention	1494.92	1485.57	1470.89	1473.37	1442.90	1425.30	1417.63
	Nutrient cycling	145.08	145.54	146.74	146.76	145.35	146.20	147.98
	Biodiversity	1105.92	1069.61	1059.48	1058.50	1036.99	1039.02	1009.11
Culture	Aesthetic landscape	532.50	513.50	510.40	509.79	500.55	501.91	482.42
Total		14,792.87	14,204.57	14,375.90	14,347.98	14,225.63	14,300.22	13,258.89

**Table 5:** Sensitivity coefficient resulting from the adjustment of the equivalent value coefficient.

Land-Use Types	1990	1995	2000	2005	2010	2015	2020
Paddy	0.36	0.35	0.30	0.31	0.29	0.24	0.22
Dry land	0.06	0.06	0.06	0.06	0.06	0.06	0.06
Forest	0.06	0.06	0.06	0.06	0.06	0.06	0.06
Grassland	0.25	0.28	0.30	0.30	0.43	0.44	0.65
Water area	0.77	0.81	0.79	0.80	0.78	0.76	0.82
Unused land	0.01	0.01	0.01	0.01	0.01	0.01	0.01

## Data Availability

The data presented in this study are available upon request from the corresponding author. The data are not publicly available due to privacy or other restrictions.
